# Diagnostic Performance and Safety of Endoscopic Ultrasound-Guided Fine-Needle Biopsy (EUS-FNB) Under Conscious Sedation for Small Solid Pancreatic Lesions (<20 mm): A Retrospective Cohort Study

**DOI:** 10.3390/jcm15218522

**Published:** 2026-11-02

**Authors:** Stefano Landi, Francesca Lodato, Paquale Apolito, Cecilia Fiorini, Emanuele Dabizzi, Nicolò Mezzina, Giacomo Tamanini, Stefania Ghersi, Marco Bassi, Adele Fornelli, Vincenzo Cennamo

**Affiliations:** 1 Department of Gastroenterology and Interventional Endoscopy, AUSL Bologna Bellaria, Maggiore Hospital, 40133 Bologna, Italy; stefano.landi@ausl.bologna.it (S.L.); pasquale.apolito@ausl.bologna.it (P.A.); giacomo.tamanini@ausl.bologna.it (G.T.); stefania.ghersi@ausl.bologna.it (S.G.); marzo.bassi@ausl.bologna.it (M.B.); vincenzo.cennamo@ausl.bologna.it (V.C.); 2 Digestive Endoscopy Unit, Gastroenterology and Endoscopy, Fondazione IRCCS Ca’ Granda Ospedale Maggiore Policlinico, 20122 Milan, Italy; emanuele.dabizzi@policlinico.mi.it; 3 Paoli-Calmettes Institute, Department of Hepatogastroenterology, 13273 Marseille, France; mezzinan@ipc.unicancer.it; 4 Pathology Unit, Ospedale Maggiore "C.A. Pizzardi", AUSL Bologna, 40133 Bologna, Italy; adele.fornelli@ausl.bo.it

**Keywords:** EUS, FNB, conscious sedation

## Abstract

**Background/Objectives:** The diagnostic evaluation of small (<20 mm) solid pancreatic lesions (SPLs) remains technically challenging, and most available EUS-FNB data derive from cohorts performed under deep sedation or heterogeneous sedation protocols. This study evaluated the diagnostic performance, feasibility, and safety of endoscopic ultrasound-guided fine-needle biopsy (EUS-FNB) performed under standard conscious sedation in consecutive eligible patients with SPLs < 20 mm. **Methods:** This was a single-center retrospective analysis of a prospectively maintained database including 114 patients with SPLs < 20 mm who underwent EUS-FNB under conscious sedation between January 2021 and December 2024. Procedural characteristics, histologic adequacy, diagnostic accuracy, repeated procedures, and adverse events were collected. Index diagnostic accuracy was calculated on completed index procedures; procedures aborted because of intolerance were analyzed as sedation failures and included in the safety and cumulative analyses. Variables associated with tissue core adequacy were assessed in an exploratory univariate analysis. **Results:** The mean target lesion size was 14.2 ± 4.3 mm, and 31 lesions (27.2%) were sub-centimetric (<10 mm). A 22-gauge needle was used in 98.2% of cases, a Franseen-tip needle in 80.4%, and at least two passes were performed in 87.5%. Among the 112 completed index procedures, histologic adequacy was 93.8% (105/112, 95% CI 87.7–96.9) and diagnostic accuracy was 91.1% (102/112; 95% CI 84.3–95.1), increasing to a cumulative accuracy of 97.4% (111/114) after targeted repeat procedures. Index accuracy was 93.5% (29/31) for lesions < 10 mm and 90.1% (73/81) for lesions of 10–19 mm (*p* = 0.724). Counting the three repeat procedures performed with anesthesiology support as failures of the conscious sedation pathway, cumulative accuracy under conscious sedation alone was 94.7% (108/114). Sensitivity, specificity, PPV and NPV of the index EUS-FNB for malignancy were 92.5%, 100%, 100% and 73.1%, respectively. No severe intraprocedural adverse events occurred; two index procedures (1.8%) were aborted because of intolerance under conscious sedation and required anesthesiology support, and two mild, self-limiting cases of acute pancreatitis (1.8%) occurred after the procedure. In the exploratory analysis, performing at least two needle passes was the only variable associated with sample adequacy (*p* = 0.041), although this finding was based on only seven inadequate samples. **Conclusions:** EUS-FNB under conscious sedation appears feasible, with high diagnostic yield and a low rate of adverse events, in consecutive eligible patients with small SPLs, including sub-centimetric targets. These findings support a pragmatic real-life workflow based on careful patient selection, adequate monitoring, modern FNB needles, macroscopic core assessment, and consideration of at least two passes whenever feasible (a hypothesis-generating finding), with ROSE or anesthesiologist support reserved for selected unresolved or poorly tolerated cases. Prospective comparative studies are needed before concluding equivalence with propofol-based deep sedation or systematic anesthesiologist-directed care.

## 1. Introduction

Endoscopic ultrasound-guided tissue acquisition (EUS-TA) is currently considered the reference minimally invasive technique for sampling solid pancreatic lesions (SPLs). Recent guideline documents have progressively emphasized the role of fine-needle biopsy (FNB), particularly with end-cutting needles, because it provides tissue architecture, histologic cores, and material suitable for immunohistochemistry and ancillary testing [1,2]. In recent years, the transition from fine-needle aspiration (FNA) to FNB has therefore changed the diagnostic pathway of pancreatic masses, especially in centers where rapid on-site cytological evaluation (ROSE) is not routinely available [1,2].

Despite these technical advancements, the pathological diagnosis of small SPLs remains a relevant challenge. The widespread use and refinement of cross-sectional imaging have increased the incidental detection of small SPLs, including sub-centimetric lesions [2]. However, diagnostic performance may decrease as lesion size decreases, because of reduced target stability, a narrower puncture window, interposed vessels or pancreatic duct, and the limited amount of retrievable tissue [3,4,5,6,7]. In this setting, optimization of needle type, number of passes, sampling technique, and specimen triage becomes crucial to maximize diagnostic yield while maintaining procedural safety.

EUS-TA is an invasive procedure requiring adequate sedation and careful patient monitoring. While deep sedation with propofol is frequently used for complex EUS procedures, anesthesiologist availability, local regulations, costs, and patient-related risk factors may limit its systematic use. Conversely, previous studies and guidelines suggest that conscious/moderate sedation can be acceptable for diagnostic EUS and selected EUS-TA procedures, whereas deep sedation or general anesthesia should be considered for therapeutic EUS, combined EUS-ERCP, highly complex procedures, or high-risk patients [8,9,10,11].

Most available data on EUS under moderate sedation derive from diagnostic EUS or EUS-FNA series, often compared with propofol in heterogeneous populations [9,10]. Conversely, data on EUS-FNB with modern end-cutting needles performed exclusively under conscious sedation, and specifically in small SPLs, remain scarce.

The primary objective of this study was to evaluate the diagnostic accuracy of EUS-FNB performed under standard conscious sedation specifically for small SPLs measuring strictly less than 20 mm. Secondary aims included assessing feasibility (rate of procedures aborted for intolerance), safety, histologic adequacy, evaluating the clinical role of repeated procedures after an initial non-diagnostic attempt, and exploring clinical, anatomical, and technical factors associated with tissue core adequacy.

## 2. Materials and Methods

### 2.1. Study Design and Patient Population

This was a single-center retrospective analysis of a prospectively maintained database including patients who underwent EUS-FNB for solid pancreatic lesions (SPLs) at our institution (Maggiore Hospital, Bologna, Italy).

Patients who underwent EUS- guided tissue sampling (FNB or FNA) of pancreatic lesions between January 2021 and December 2024 were screened. The inclusion criteria were: (1) adult patients (≥18 years of age); (2) presence of an SPL measuring < 20 mm on pre-procedural imaging; and (3) EUS-FNB performed as the primary sampling modality under conscious sedation. Patients were excluded if they had predominantly cystic lesions, uncorrectable coagulopathy, or if they underwent the procedure under deep sedation/propofol as the primary initial approach. Patients whose index procedure was started under conscious sedation but aborted because of intolerance were not excluded: they were included in the safety, feasibility and cumulative analyses, but not in the calculation of index diagnostic accuracy, which was restricted to completed procedures.

At our center, conscious sedation is the standard approach for EUS-FNB, whereas anesthesiology assistance is reserved for patients with severe cardiorespiratory or neurological disease. All 114 eligible patients with SPLs < 20 mm were included consecutively during the study period; no patient with a small solid lesion underwent FNA alone or received primary anesthesiologist-directed sedation.

The study was conducted according to the procedures provided for by current legislation in terms of data confidentiality and compliance with ethical requirements and it was approved by the institutional review board (Comitato Etico di Area Vasta Emilia Centro della Regione Emilia-Romagna, CE-AVEC registration number: CE 226-2025-OSS-AUSLBO-25034) and complied with the provisions of the Good Clinical Practice guidelines and the Declaration of Helsinki.

For patients followed up at our hospital, Informed Consent was obtained as part of one of the visits included in the normal course of care. For deceased or untraceable patients, based on the General Authorization for the Processing of Personal Data Performed for Scientific Research Purposes issued by the Italian Privacy Guarantor (No. 72 March 2012), personal data were processed in the absence of consent.

### 2.2. Endoscopic Procedure and Sedation Protocol

All primary EUS-FNB procedures were performed by endosonographers with different levels of experience (≤5 or >5 years) in this technique.

Overall, four endosonographers performed the index procedures. Standard conscious sedation (sedo-analgesia) was administered utilizing a combination of intravenous Midazolam and Meperidine, titrated to achieve adequate patient comfort while maintaining spontaneous ventilation and airway reflexes. Continuous pulse oximetry and heart rate monitoring, together with non-invasive blood pressure measurements, were maintained throughout the procedure; capnography was not routinely used. For this retrospective analysis, clinically significant desaturation was defined as SpO_2_ < 90% and clinically significant hypotension as systolic blood pressure < 90 mmHg. These numerical thresholds have also been employed in a prospective study of sedation-related events during gastrointestinal endoscopy [12]. According to routine clinical practice, desaturation is managed with supplemental oxygen via nasal cannulae (1–6 L/min, titrated as needed) and hypotension with intravenous normal saline when clinically indicated. After the procedure, all patients were monitored in the recovery room for at least 1 h.

When adequate tolerance could not be achieved under conscious sedation, the procedure was aborted and sampling was subsequently performed with anaesthesiological support.

EUS examinations were conducted using a linear array echoendoscope (EG-3870UTK, Pentax Medical, Tokyo, Japan). Once the target lesion was identified and evaluated (including anatomical location and precise size measurement), EUS-FNB was performed. The choice of needle type, needle gauge, and number of passes was left to the operator’s discretion. All samples were obtained with the slow-pull technique, and the fanning technique was routinely applied in all procedures. The puncture area was selected according to the technical feasibility of the biopsy, avoiding interposition of vessels, the main pancreatic duct or the common bile duct along the needle path.

Data regarding the type of biopsy needle utilized, both in terms of diameter (gauge) and needle tip geometry, as well as the number of passes performed, were collected for analysis. The FNB needles used were Acquire (Boston Scientific, Marlborough, MA, USA), considered a Franseen-tip needle, and Trident (Micro-Tech, Nanjing, China), SharkCore (Covidien/Medtronic, Minneapolis, MN, USA), ProCore (Cook Medical, Bloomington, IN, USA) and EZ Shot 3 Plus (Olympus, Tokyo, Japan), considered non-Franseen needles.

For patients requiring a second EUS-FNB procedure due to a non-diagnostic sample or procedure intolerance to conscious sedation, the procedure was repeated as directed by the endoscopist, once again under sedation and analgesia or under anesthetic supervision, with the option of adding rapid on-site evaluation (ROSE) performed by an experienced cytopathologist, always in agreement with the professional performing the procedure.

### 2.3. Pathological Evaluation and Diagnostic Standard

Macroscopic on-site evaluation (MOSE) was performed by the endoscopist on each pass during the index procedures; a sample was considered macroscopically adequate when a visible core ≥ 10 mm was obtained. All collected specimens were then fixed in formalin and processed for definitive histopathological and immunophenotypic evaluation. The adequacy of the FNB sample and the final histopathological diagnosis were evaluated by an expert pathologist not blinded to clinical data according to standardized pancreaticobiliary reporting principles and the World Health Organization framework for pancreaticobiliary cytopathology [13]. Samples classified as atypical or suspicious for malignancy would have been considered non-diagnostic. Concordance between the FNB result and the final diagnosis was defined as agreement in the histotype of the neoplasm and, for NETs in operated patients, also in tumor grade; discordance was defined as a different histotype or grade on the surgical specimen, or a different benign/malignant category.

The reference gold standard for the final diagnosis was based on: (1) definitive histological evaluation of the surgical specimen for patients who subsequently underwent surgical resection; or (2) a minimum of 6 months of clinical and radiological follow-up for non-operated patients, assessing for disease progression (in malignant lesions) or stability (in benign conditions). In non-operated patients, the final diagnosis was the FNB diagnosis, unless different findings emerged during follow-up (e.g., evolution of a lesion with a benign FNB result into a malignancy). Follow-up was based on computed tomography (CT), magnetic resonance imaging (MRI) or EUS.

### 2.4. Outcomes and Definitions

The primary outcome was the diagnostic accuracy of the index EUS-FNB performed under conscious sedation, defined as the proportion of completed index procedures in which the EUS-FNB result correctly matched the final gold-standard diagnosis (surgery or follow-up). Inadequate samples obtained at completed index procedures were conservatively classified as non-diagnostic and discordant with the final diagnosis. Similarly, samples classified as ‘atypical’ or ‘suspicious for malignancy’ according to the WHO Reporting System [13] were considered non-diagnostic and, therefore, discordant with the final diagnosis (conservative approach). The two index procedures aborted because of patient intolerance were excluded from the calculation of index diagnostic accuracy and classified as failures of the conscious sedation pathway. We calculated diagnostic accuracy for completed index procedures and cumulative diagnostic accuracy after repeat EUS-FNB. Three repeat procedures required anesthesiology support: two following intolerance-related interruption of the index procedure and one involving a technically challenging subcentimetric lesion, for which ROSE was also employed. Therefore, to distinguish the diagnostic performance of the conscious sedation strategy from that of the overall diagnostic pathway, cumulative accuracy was also calculated for conscious sedation alone, counting all three cases requiring anaesthesiology support as failures of this pathway.

Index adequacy and accuracy were also reported separately for lesions < 10 mm and 10–19 mm. Sensitivity, specificity, positive predictive value (PPV) and negative predictive value (NPV) of the index EUS-FNB for malignancy were calculated considering as malignant PDAC, NETs, renal cell carcinoma metastases, degenerated IPMN/high-grade dysplasia and gastrointestinal stromal tumors (GIST), mixed PDAC–NET, and as benign inflammatory lesions, accessory spleen and serous cystadenoma; inadequate results were considered negative. As a sensitivity analysis, the same parameters were also calculated on histologically adequate index samples only. Secondary outcomes included feasibility (rate of procedures aborted for intolerance), sample adequacy (macroscopic and histologic) and the exploration of clinical, anatomical, and technical factors associated with tissue core adequacy. Safety was assessed by recording the incidence and severity of intraprocedural and post-procedural adverse events (bleeding, perforation, pancreatitis and sedation-related events), categorized according to the American Society for Gastrointestinal Endoscopy (ASGE) lexicon [14].

### 2.5. Statistical Analysis

Data were collected and organized into a dedicated database. Continuous variables were expressed as mean ± SD and range. Categorical variables were reported as absolute frequencies (*N*) and relative percentages (%). 95% confidence intervals (CI) for accuracy, adequacy, sensitivity, specificity, PPV and NPV were calculated with the Wilson method. Index accuracy in lesions < 10 mm and 10–19 mm was compared with Fisher’s exact test. Follow-up duration was expressed as median and interquartile range (IQR). To explore variables associated with tissue core adequacy, an exploratory univariate analysis was performed comparing patient-related and technique-related features, including age, weight, ASA status, lesion size category, anatomical location, needle caliber, tip geometry, operator experience, and sampling intensity, between histologically inadequate and adequate samples. Given the small number of inadequate samples (*n* = 7), multivariable modeling was not performed and the analysis was considered hypothesis-generating.

Continuous variables were compared between the two independent groups using the non-parametric Mann–Whitney U test. For the comparison of categorical variables, Pearson’s Chi-square test or Fisher’s exact test (with the Fisher-Freeman-Halton extension for tables larger than 2 × 2) was applied, as appropriate. A two-tailed *p*-value < 0.05 was considered statistically significant. Statistical analyses were performed using SPSS software version 29 (IBM SPSS Statistics, IBM Corp., Armonk, NY, USA).

## 3. Results

### 3.1. Study Population and Baseline Characteristics

Of the 603 patients who underwent EUS-guided tissue sampling (FNB or FNA) of pancreatic lesions during the study period, 489 were excluded: 366 had solid lesions ≥ 20 mm and 123 had predominantly cystic lesions. No patient with a small solid pancreatic lesion underwent FNA alone.

**Figure 1:**
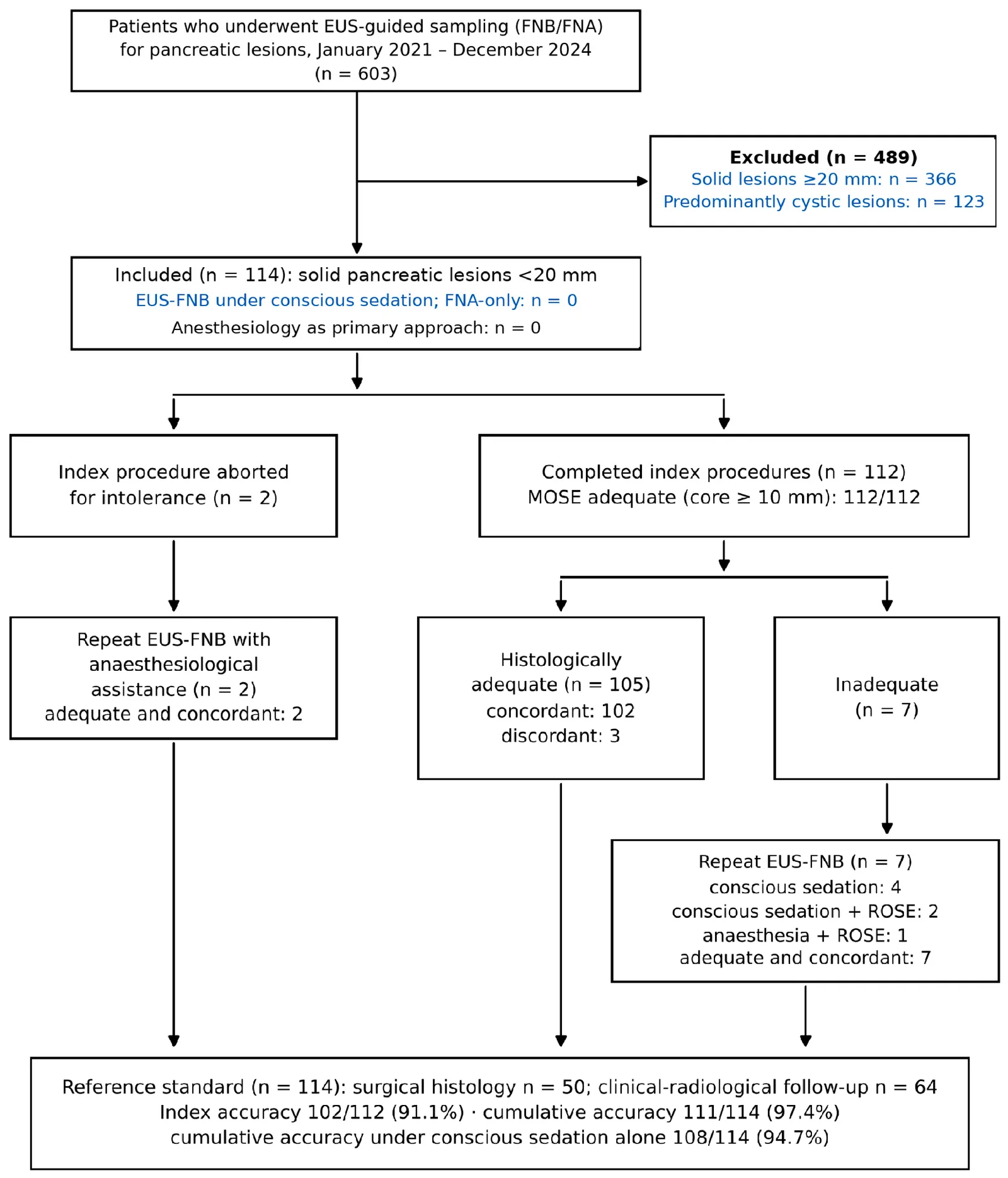
Flow diagram of patient selection and diagnostic pathway. MOSE, macroscopic on-site evaluation; ROSE, rapid on-site evaluation.

A total of 114 patients presenting with small (<20 mm) solid pancreatic lesions (SPLs) who underwent Endoscopic Ultrasound-guided Fine-Needle Biopsy (EUS-FNB) were included in the analysis. The patient selection process is summarized in the flow diagram (Figure 1).

The study cohort comprised 60 female patients (52.6%) and 54 male patients (47.4%). The overall mean age of the population at the time of the procedure was 66.9 ± 12.4 years, spanning an age range from 26.1 to 90.3 years. The mean body weight of the analyzed subjects was 76.5 ± 15.7 kg. Regarding conscious sedation parameters administered during the endoscopic interventions, the mean delivered dose of Meperidine was 95.0 ± 22.6 mg, while the mean dose of Midazolam reached 4.9 ± 1.3 mg.

The morphological and technical baseline features highlighted a mean target lesion dimension of 14.2 ± 4.3 mm, with a range between 5.0 and 19.0 mm. Thirty-one lesions (27.2%) were sub-centimetric (<10 mm). Anatomically, the lesions were distributed across the pancreatic segments as follows: 38 (33.3%) were located within the pancreatic head, 4 (3.5%) within the uncinate process, 42 (36.8%) in the pancreatic body, and 30 (26.3%) within the tail. Of the 114 index procedures, 60 (52.6%) were performed by endosonographers with ≤5 years of experience and 54 (47.4%) by endosonographers with >5 years of experience.

Technical data refer to the 112 completed index procedures. A 22-gauge needle was used in most cases (98.2%, *n* = 110), whereas a 20-gauge caliber was used in only 2 instances (1.8%). A third-generation end-cutting Franseen needle tip design was employed in 80.4% (*n* = 90) of the procedures, while non-Franseen geometries were selected for the remaining 22 cases (19.6%). In terms of sampling execution, at least two needle passes were successfully performed in 87.5% (*n* = 98) of the patient population. Definitive surgical resection was subsequently undertaken in 50 patients (43.9%), whereas the remaining 64 patients (56.1%) underwent clinical and radiological follow-up management. Comprehensive data for the quantitative and qualitative baseline population characteristics are systematically categorized in Table 1 and Table 2. In the 64 non-operated patients, the median follow-up was 13.5 months (IQR 8–23; range 6–50), and follow-up was based mainly on CT (*n* = 32), EUS (*n* = 30) or MRI (*n* = 2).

### 3.2. Diagnostic Accuracy and Safety Outcomes

Histological adequacy of tissue cores obtained during the index procedure was achieved in 93.8% of completed index procedures (105/112; 95% CI 87.7–96.9). MOSE showed a macroscopically adequate core (≥10 mm) in all 112 completed index procedures; therefore, 7 of the 112 MOSE-adequate samples (6.3%) were histologically inadequate/non-diagnostic. No sample was classified as atypical or suspicious for malignancy.

A subgroup of 9 patients required repeated EUS-FNB because of a histologically inadequate/non diagnostic sample (*n* = 7) or intolerance (*n* = 2) during the initial index procedure. To maximize diagnostic yield during the repeat procedure, technical and organizational modifications were implemented according to the clinical scenario, including anesthesiology assistance and/or ROSE when available. After targeted secondary procedures in patients with an initial non-diagnostic or suboptimal result, the cumulative final diagnostic accuracy increased to 97.4% (111/114; 95% CI 92.5–99.1). Three of the nine repeat procedures were performed with anesthesiologist support: two following index procedures aborted because of patient intolerance and one following inadequate tissue sampling of a technically challenging subcentimetric lesion, for which ROSE was also performed during the repeat procedure. Counting these three cases as failures of the conscious sedation pathway, cumulative accuracy under conscious sedation alone was 94.7% (108/114; 95% CI 89.0–97.6). Therefore, the cumulative accuracy reflects the whole diagnostic pathway and not conscious sedation alone. The diagnostic accuracy outcomes are presented in Table 3.

The most frequent diagnosis was pancreatic neuroendocrine tumor (NET), accounting for 41.0% (*n* = 43) of adequate index samples, closely followed by pancreatic ductal adenocarcinoma (PDAC), which was identified in 33.3% (*n* = 35) of cases. The remaining benign and malignant diagnostic findings comprised chronic lymphocytic or fibrotic inflammation (9.5%, *n* = 10), renal cell carcinoma metastases (6.7%, *n* = 7), active acute inflammation (4.8%, *n* = 5), accessory intrapancreatic spleens (2.9%, *n* = 3), degenerated/high-grade intraductal papillary mucinous neoplasm (IPMN) (1.0%, *n* = 1), and solid variants of serous cystadenoma (1.0%, *n* = 1).

Considering the 112 completed index procedures, with inadequate samples counted as non-diagnostic, the diagnostic accuracy of the index EUS-FNB was 91.1% (102/112; 95% CI 84.3–95.1). Counting the two procedures aborted for intolerance as failures, intention-to-diagnose index accuracy was 89.5% (102/114; 95% CI 82.5–93.9). Final diagnoses according to the reference standard are reported in Table 4. Index accuracy was 93.5% (29/31; 95% CI 79.3–98.2) for lesions < 10 mm and 90.1% (73/81; 95% CI 81.7–94.9) for lesions of 10–19 mm (*p* = 0.724); histologic adequacy was 96.8% (30/31; 95% CI 83.8–99.4) and 92.6% (75/81; 95% CI 84.8–96.6), respectively. After repeat procedures, cumulative accuracy was 96.8% (30/31) for lesions < 10 mm and 97.6% (81/83) for lesions of 10–19 mm. The two additional patients included in the cumulative analysis had lesions measuring 10–19 mm and underwent repeat EUS-FNB following index procedures aborted for intolerance. For the diagnosis of malignancy, the index EUS-FNB had a sensitivity of 92.5% (86/93; 95% CI 85.3–96.3), a specificity of 100% (19/19; 95% CI 83.2–100), a PPV of 100% (86/86; 95% CI 95.7–100) and an NPV of 73.1% (19/26; 95% CI 53.9–86.3). Six of the seven false-negative results were inadequate samples, conservatively classified as negative, and no false-positive result was observed. When the analysis was restricted to the 105 histologically adequate index samples, sensitivity, specificity, PPV and NPV for malignancy were 98.9% (86/87; 95% CI 93.8–99.8), 100% (18/18; 95% CI 82.4–100), 100% (86/86; 95% CI 95.7–100) and 94.7% (18/19; 95% CI 75.4–99.1), respectively. Diagnostic performance according to lesion size is reported in Table 5.

The ten discordant index procedures comprised seven inadequate samples and three adequate samples whose FNB result did not match the final diagnosis. All seven inadequate samples were resampled and yielded adequate specimens concordant with the final diagnosis (PDAC, *n* = 3; NET, *n* = 2; GIST, *n* = 1; chronic inflammation, *n* = 1). Among the three adequate but discordant samples, two concerned the histological subtype or grade of a malignant lesion: a NET G1 at FNB was graded as NET G2 on the surgical specimen (9 mm, pancreatic body), and a PDAC at FNB was diagnosed as a mixed PDAC–NET neoplasm on the surgical specimen (13 mm, pancreatic tail). In the third case, a lesion with chronic inflammation at index FNB (18 mm, pancreatic head) was managed with follow-up, and a repeat EUS-guided biopsy during follow-up showed a NET G1; this was the only false-negative result among adequate samples. None of these three patients underwent a repeat procedure during the initial diagnostic work-up (in the third case, the NET was diagnosed at a biopsy performed during follow-up); therefore, all three remained discordant in the cumulative analysis.

Of the 19 adequate index samples with a benign FNB result (chronic inflammation, *n* = 10; acute inflammation, *n* = 5; accessory spleen, *n* = 3; solid serous cystadenoma, *n* = 1), 6 were verified by surgical histology (chronic inflammation, *n* = 5; acute inflammation, *n* = 1) and 13 by clinical and radiological follow-up (chronic inflammation, *n* = 5; accessory spleen, *n* = 3; acute inflammation, *n* = 4; serous cystadenoma, *n* = 1), with a median follow-up of 20.5 months (range 6–50); the three accessory spleens were followed up with CT. All surgically resected benign lesions were confirmed, whereas one of the 13 lesions followed up (chronic inflammation) was re-classified as NET G1 at follow-up biopsy.

From a safety standpoint, no severe intraprocedural adverse events were recorded. The index procedure was aborted in 2 of 114 patients (1.8%) because of intolerance under conscious sedation; both patients subsequently underwent sampling with anesthesiology support and were therefore excluded from the index accuracy analysis (*n* = 112). In both cases, the repeat procedure yielded an adequate sample, with a final diagnosis of PDAC. Post-procedural adverse events occurred after 2 of the 112 completed index procedures (1.8%) and consisted of mild, self-limiting acute pancreatitis. Both patients with post-procedural pancreatitis required hospitalization. No sedation-related intraprocedural adverse event was recorded: no clinically significant oxygen desaturation or hypotension was observed, and no reversal agents were required. All patients were monitored in the recovery room for at least 1 h after the procedure.

### 3.3. Technical Specifications and Outcomes of Repeated Procedures

A subgroup of 9 patients required the execution of a repeated EUS-FNB procedure due to inadequate tissue acquisition during the index procedure (*n* = 7) or intolerance to conscious sedation (*n* = 2). At repeat procedures, anesthesiologist-directed sedation was used in 33.3% of cases, and Rapid On-Site Evaluation (ROSE) was utilized in 33.3% to guide immediate core acquisition. A 22-gauge needle was used in all 9 repeat procedures. Franseen tip geometries were applied in 55.6% (*n* = 5) of these repeated samplings, and non-Franseen end-cutting needles were selected in the remaining 44.4% (*n* = 4). In all repeat procedures, at least 2 passes were performed. All repeat procedures yielded histologically adequate samples. The data regarding the repeated procedures are listed in Table 6.

### 3.4. Univariate Predictor Analysis for Sample Adequacy

To identify variables associated with successful tissue core acquisition, a univariate statistical analysis compared patient-related and technique-related features between histologically inadequate (*n* = 7) and adequate samples (*n* = 105).

Age (70.9 ± 6.5 vs. 66.8 ± 12.7 years; *p* = 0.457) and total body weight (75.9 ± 14.5 vs. 76.1 ± 15.5 kg; *p* = 0.928) demonstrated no statistical association with technical outcome. Sub-centimetric size was not associated with inadequacy: lesions under 10 mm represented 14% of the inadequate cohort compared to 29% of the adequate group (*p* = 0.671). Similarly, ASA physical status (*p* = 0.687) and specific anatomical location within the pancreatic segments (*p* = 0.333) were not significantly associated with inadequacy. Furthermore, technical parameters such as needle size caliber (*p* = 1.000), operator experience status (*p* = 0.115), and needle tip geometry (Franseen vs. non-Franseen designs; *p* = 1.000) were not significantly associated with inadequacy, although the small number of inadequate samples limits statistical power; notably, 6 of 7 inadequate samples were obtained by endosonographers with ≤ 5 years of experience (*p* = 0.115).

In this exploratory analysis, the number of needle passes was the only variable significantly associated with tissue core adequacy. Fewer than two passes were associated with core inadequacy, accounting for 43% of inadequate samples compared with only 10% of adequate samples (*p* = 0.041). Conversely, 90% of adequate samples were obtained with two or more passes. Given the small number of events, this finding should be considered hypothesis-generating. A single pass was performed in 14 patients at the endoscopist’s discretion (e.g., satisfactory MOSE and/or technical difficulty).

The full univariate analysis is presented in Table 7.

## 4. Discussion

This single-center retrospective analysis of a prospectively maintained database evaluated the performance of EUS-FNB under standard conscious sedation in a consecutive cohort of eligible patients with small (<20 mm) solid pancreatic lesions. To our knowledge, data on EUS-FNB of small SPLs performed exclusively under benzodiazepine–opioid conscious sedation are scarce. The main findings are clinically relevant: first, EUS-FNB performed under conscious sedation was feasible, with only 2 procedures (1.8%) aborted for intolerance, and achieved high histologic sample adequacy despite the small size of the targets; second, the index diagnostic accuracy was high and increased further after a structured rescue pathway with repeated procedures; third, adverse events and sedation-related intolerance were uncommon; and fourth, the number of needle passes was the only variable significantly associated with sample adequacy in an exploratory univariate analysis.

The diagnostic work-up of small SPLs is increasingly important because modern cross-sectional imaging detects pancreatic nodules at earlier stages and in asymptomatic patients. However, small lesions remain technically demanding targets. Reduced lesion diameter may limit needle maneuverability and fanning, increase the impact of respiratory motion and scope instability, and reduce the amount of retrievable core tissue. This is reflected in the previous literature: Conti Bellocchi et al. reported higher diagnostic accuracy and sample adequacy with FNB than FNA in SPLs ≤ 15 mm [4], whereas Takahashi et al. showed that the accuracy of histological diagnosis using a Franseen needle was lower for lesions < 10 mm than for larger lesions [5]. Lai et al. further demonstrated that smaller tumor size and tumor type influence macroscopic tissue core retrieval [6], and Sun et al. identified lesion size, needle type, and number of passes among factors associated with diagnostic performance and adequacy [7]. Against this background, the high adequacy observed in our cohort suggests that FNB can maintain strong diagnostic performance even in a small-lesion setting when performed by endosonographers using an adequate sampling strategy. Notably, index accuracy in sub-centimetric lesions was similar to that observed in lesions of 10–19 mm (93.5% vs. 90.1%; *p* = 0.724), although the wide confidence intervals limit the precision of this comparison.

However, neuroendocrine tumors accounted for 41% of diagnoses in our series; as these lesions are usually well demarcated and less desmoplastic than PDAC, this case mix may have favored tissue acquisition and should be considered when comparing our results with PDAC-predominant series.

Our findings are also consistent with the current shift from FNA to FNB for pancreatic tissue acquisition. Recent ASGE guidance supports the use of FNB needles over FNA needles for solid pancreatic masses [1], and the ESGE technical review emphasizes the role of end-cutting FNB needles for tissue acquisition in solid pancreatic lesions [2]. Meta-analyses and comparative studies have reported improved or at least comparable diagnostic performance with FNB, together with advantages in histologic core procurement, tissue architecture preservation, and suitability for ancillary testing [15,16,17,18]. In this context, the high use of 22-gauge FNB needles and the predominance of Franseen needle design in our series are aligned with contemporary practice [19,20,21,22].

The number of passes deserves specific consideration. In our cohort, performing at least two passes was the only variable significantly associated with sample adequacy at univariate analysis. Notably, a single pass was performed at the endoscopist’s discretion, including cases with a satisfactory MOSE, yet 3 of these 14 samples were histologically inadequate. Since MOSE was judged adequate in all completed index procedures, whereas 7 samples (6.3%) were histologically inadequate, MOSE alone may overestimate histologic adequacy in small lesions. However, the association between number of passes and adequacy is based on very few inadequate samples (3 after a single pass vs. 4 after two or more passes) and should be regarded as hypothesis-generating. This finding is in line with the prospective multicenter study by Mangiavillano et al. [3], which showed a significant increase in diagnostic accuracy and sample adequacy from the first to the second pass with a 22G Franseen needle, without additional benefit from a third pass in that protocol. A recent systematic review and meta-analysis also suggested that two passes with Franseen or fork-tip needles, and three passes with any FNB needle, may be sufficient to achieve optimal diagnostic performance [23]. Therefore, in small SPLs, performing at least two passes whenever technically feasible may be a reasonable pragmatic option, pending confirmation in larger studies, while individualizing the need for further passes according to MOSE, target characteristics, bleeding risk, lesion location, and clinical suspicion [24,25].

The role of MOSE and ROSE should be interpreted in a practical, real-life perspective. ROSE can improve adequacy assessment but is not available in many centers and may increase procedure time and organizational complexity. Conversely, MOSE is immediately available to the endoscopist and has gained increasing support in the FNB era [26,27]. In our study, the primary procedures were performed using macroscopic assessment of tissue cores, while ROSE was reserved for selected repeated procedures. This approach may represent a reasonable workflow for centers without systematic cytopathologist availability, particularly when modern FNB needles are used and at least two passes are performed.

A distinctive aspect of this study is the systematic use of conscious sedation for the index procedure. Most published EUS-FNB studies include deep sedation, anesthesiologist-administered propofol, or heterogeneous sedation regimens, whereas our cohort specifically evaluates a conscious sedation pathway. Our data should not be interpreted as proving equivalence between conscious sedation and propofol-based deep sedation, because no parallel control group was included. However, they suggest that, in selected patients and within a standardized workflow, EUS-FNB of small SPLs under conscious sedation can achieve technical success and diagnostic yield in the range reported for series performed under deep sedation. This is consistent with the concept that diagnostic EUS and selected EUS-guided sampling procedures may be performed with moderate sedation, while complex therapeutic EUS, combined EUS-ERCP procedures, or high-risk patients may require deep sedation or anesthesiological support [8,9,10,11].

These findings are particularly relevant when interpreted in the context of contemporary European and Italian sedation practice. In the recent ESGE survey on sedation practices in gastrointestinal endoscopy, sedation choices varied widely according to endoscopists’ and patients’ preferences, cultural attitudes, healthcare resource availability, local policies, and national legislation; propofol administration remained restricted to anesthesiology personnel in more than 60% of respondent settings, while benzodiazepine-opioid regimens were still reported as a relevant option for EUS procedures [28]. The Italian multicentric Emilia-Romagna observational study by Manno et al. similarly highlighted marked heterogeneity in anesthesiologist-directed care (ADC) for elective gastrointestinal endoscopy, with potential overuse of ADC for diagnostic procedures and underuse for complex therapeutic procedures [29]. Taken together, these data support a selective and resource-conscious sedation strategy. Conscious sedation should not be viewed as a substitute for anesthesiology support when patient-related risk factors or procedural complexity require it; however, our findings suggest that a structured conscious sedation pathway for selected patients undergoing diagnostic EUS-guided FNB may be feasible and may help reserve anesthesiology resources for high-risk patients and advanced therapeutic endoscopy.

The safety profile observed in our series was favorable. The absence of severe unexpected intraprocedural complications, the low rate of intolerance-related interruption, and the low frequency of mild post-procedural adverse events support the feasibility of this approach. Nevertheless, patient selection remains essential. Obesity, higher ASA class, obstructive sleep apnea, frailty, and relevant cardiopulmonary comorbidities are recognized risk factors for sedation-related adverse events and should prompt careful pre-assessment and, when appropriate, anesthesiology support [8,9,10,11]. Moreover, adverse events should be uniformly defined and reported according to standardized lexicons, such as the ASGE adverse event classification [14].

The subgroup of patients undergoing repeated EUS-FNB is also clinically important. A non-diagnostic or inadequate first procedure can delay treatment, especially in suspected pancreatic cancer. In our cohort, a repeat procedure with technical or anesthesiology escalation allowed recovery of several initially unresolved cases, contributing to the increase in cumulative diagnostic accuracy. However, three of the nine repeat procedures were performed with anesthesiology support; therefore, the cumulative accuracy (97.4%) reflects the whole diagnostic pathway and should not be attributed to conscious sedation alone, for which cumulative accuracy was 94.7% (108/114). This finding is consistent with studies showing that repeated EUS-FNB after a first non-diagnostic or negative sampling can provide additional diagnostic yield and may identify both malignant and benign conditions [30,31]. In clinical practice, repeat sampling should be considered when the pre-test probability of malignancy remains high despite a negative or inadequate initial specimen. In our series, specificity and PPV for malignancy were 100%, whereas the NPV was 73.1%: six of the seven false-negative results were inadequate samples, and one lesion with chronic inflammation at FNB proved to be a NET G1 at follow-up biopsy. Therefore, an inadequate or benign FNB result in a small SPL should prompt repeat sampling or close follow-up.

Beyond binary diagnosis of malignancy, FNB has growing relevance for histologic characterization and precision oncology. Small SPLs include not only pancreatic ductal adenocarcinoma but also neuroendocrine tumors, metastases, inflammatory masses, accessory spleen, solid pseudopapillary neoplasms, and other uncommon entities. These diagnoses often require tissue architecture, immunohistochemistry, Ki-67 assessment, or molecular testing. Recent data suggest that EUS-FNA/FNB specimens can be adequate for next-generation sequencing in a high proportion of pancreatic malignancies [32], and the role of EUS-TA in precision medicine continues to expand [33]. Therefore, obtaining an adequate histologic core is increasingly relevant not only for diagnosis but also for treatment stratification.

This study has limitations. First, the single-center retrospective design carries a risk of selection bias and limits generalizability. Second, the absence of a direct control group undergoing propofol-based deep sedation precludes any head-to-head comparison of diagnostic yield, patient tolerance, or safety. Third, the number of patients in some subgroups, including repeated procedures and histologically inadequate samples, was small, limiting statistical power and precluding robust multivariable modeling. Fourth, adverse events and patient tolerance were based on clinical chart and endoscopy report documentation, which may underestimate minor events not systematically recorded. Fifth, although conscious sedation was the first-line approach for all patients, sedation depth was not systematically assessed using a validated scale and capnography was not routinely employed; transient periods of deeper sedation therefore cannot be excluded, particularly considering the midazolam and meperidine doses administered. The pathologist was not blinded to clinical data. Furthermore, in non-operated patients, the FNB diagnosis contributed to the final diagnosis, which may have introduced incorporation bias and overestimated accuracy. Sixth, index accuracy was calculated on completed procedures: counting the two procedures aborted for intolerance as failures, index accuracy would be 89.5% (102/114). Seventh, the reference standard for non-operated patients was based on a minimum follow-up of 6 months, which may be insufficient to exclude a missed malignancy in patients with benign FNB results, and the high proportion of neuroendocrine tumors may limit generalisability to PDAC-predominant populations. Moreover, the use of two different reference standards (surgical histology in 50 patients and clinical-radiological follow-up in 64) may have introduced differential verification bias. Finally, the predictor analysis was based on only seven inadequate samples, and its results are sensitive to the reclassification of a single case. In addition, the decision to perform a single pass was left to the endoscopist and was partly driven by technical difficulty, so the association between number of passes and adequacy may be affected by confounding by indication.

Despite these limitations, the present study provides real-world evidence supporting the feasibility, safety, and diagnostic effectiveness of EUS-FNB under conscious sedation in selected patients with small and technically challenging SPLs. The data support a pragmatic workflow based on a standardized sampling strategy, modern FNB needles, macroscopic tissue assessment, and at least two passes whenever feasible, with repeated EUS-FNB, ROSE, or anesthesiology escalation reserved for selected unresolved cases.

In conclusion, in this retrospective single-center series, EUS-FNB performed under conscious sedation was feasible in consecutive eligible patients with small solid pancreatic lesions (<20 mm), with high diagnostic accuracy and a low rate of adverse events. Diagnostic accuracy and sample adequacy were high, even in a cohort including sub-centimetric targets. In an exploratory analysis, performing at least two needle passes was associated with adequate tissue core acquisition. These findings suggest that conscious sedation EUS-FNB may be a reasonable real-life diagnostic strategy for small SPLs, provided that careful patient selection, adequate monitoring, and optimized sampling protocols are applied. However, prospective comparative studies against propofol-based sedation are needed to confirm these findings.

## Figures and Tables

**Table 1:** Demographic, clinical, and procedural parameters of the study population.

Parameter	Mean ± SD	Range (Minimum–Maximum)
Age (years)	66.9 ± 12.4	26.1–90.3
Weight (kg)	76.5 ± 15.7	44.0–124.0
Meperidine/Dose (mg)	95.0 ± 22.6	30.0–140.0
Midazolam Dose (mg)	4.9 ± 1.3	2.0–8.0
Pancreatic Lesion Size (mm)	14.2 ± 4.3	5.0–19.0

SD, standard deviation. Values are calculated across the entire primary cohort (*N* = 114).

**Table 2:** Distribution of baseline categorical variables and technical specifications.

Parameter	Stratified Category	Absolute Frequency (*N*)	Relative Frequency (%)
Gender	Female (F)	60	52.6%
	Male (M)	54	47.4%
ASA score	Class I	23	20.2%
	Class II	47	41.2%
	Class III	44	38.6%
Lesion site	Pancreatic Head	38	33.3%
	Uncinate Process	4	3.5%
	Pancreatic Body	42	36.8%
	Pancreatic Tail	30	26.3%
Sub-centimetric Lesions (<10 mm)	Present	31	27.2%
Needle Caliber Size	22G	110	98.2%
	20G	2	1.8%
Biopsy Needle Design Type	Franseen Tip Geometry	90	80.4%
	Non-Franseen Tip Geometry	22	19.6%
Number of passes	<2	14	12.5%
	≥2	98	87.5%
Primary Clinical Management	Clinical and Imaging Follow-up	64	56.1%
	Surgical Resection	50	43.9%

ASA, American Society of Anesthesiologists. Needle caliber, tip design and number of passes refer to the 112 completed index procedures.

**Table 3:** Diagnostic accuracy.

Metric	Total Evaluated Patients (*N*)	Pathologically Concordant Cases (*N*)	Accuracy (%)
First Index EUS-FNB Procedure	112	102	91.1% *
First Index EUS-FNB Procedure, Intention to diagnose	114	102	89.5%
Cumulative procedures (plus post-repeats)	114	111	97.4%
Cumulative procedures under conscious sedation alone	114	108	94.7%

EUS-FNB, endoscopic ultrasound-guided fine-needle biopsy. * Index accuracy calculated on completed index procedures (*n* = 112), with inadequate samples classified as non-diagnostic. The two procedures aborted for intolerance are included in the cumulative analysis (*n* = 114).

**Table 4:** Final diagnoses according to the reference standard (*N* = 114).

Final Diagnosis	Surgical Resection (*N*)	Clinical-Radiological Follow-Up (*N*)	Total (*N*)
Pancreatic ductal adenocarcinoma (PDAC)	23	16	39
Neuroendocrine Tumor	17	29	46
Mixed PDAC–NET neoplasm	1	0	1
Renal cell carcinoma metastasis	2	5	7
Gastrointestinal stromal tumor	0	1	1
Degenerated/high grade IPMN	1	0	1
Serous cystadenoma, solid variant	0	1	1
Chronic inflammation	5	5	10
Acute inflammation	1	4	5
Accessory spleen	0	3	3
Total	50	64	114

IPMN, intraductal papillary mucinous neoplasm; PDAC, pancreatic ductal adenocarcinoma; NET, neuroendocrine tumor.

**Table 5:** Diagnostic performance of the index EUS-FNB for malignancy in completed index procedures (*n* = 112).

Lesion Size	Sensitivity % (*n*/*N*; 95% CI)	Specificity % (*n*/*N*; 95% CI)	PPV % (*n*/*N*; 95% CI)	NPV % (*n*/*N*; 95% CI)
All lesions (*n* = 112)	92.5 (86/93; 85.3–96.3)	100 (19/19; 83.2–100)	100 (86/86; 95.7–100)	73.1 (19/26; 53.9–86.3)
<10 mm (*n* = 31)	96.2 (25/26; 81.1–99.3)	100 (5/5; 56.6–100)	100 (25/25; 86.7–100)	83.3 (5/6; 43.6–97.0)
10–19 mm (*n* = 81)	91.0 (61/67; 81.8–95.8)	100 (14/14; 78.5–100)	100 (61/61; 94.1–100)	70.0 (14/20; 48.1–85.5)

PPV, positive predictive value; NPV, negative predictive value; CI, confidence interval (Wilson method). Inadequate samples were classified as negative. Malignant: PDAC, NET, mixed PDAC–NET, renal cell carcinoma metastasis, degenerated/high-grade IPMN.

**Table 6:** Technical features of repeated procedures (*n* = 9).

		Absolute Frequency (*N*)	Relative Frequency (%)
Procedure features	Conscious sedation alone	4	44.4%
	Conscious sedation with ROSE	2	22.2%
	Anesthesiology Assistance Care Only	2	22.2%
	Anesthesiology Assistance with ROSE	1	11.1%
Needle Size	22G	9	100.0%
Biopsy Needle Core Tip Design	Franseen Tip Geometry	5	55.6%
	Non-Franseen Tip Geometry	4	44.4%
No. of passes	≥2 passes	9	100.0%
Adequacy	-	9	100.0%

ROSE, rapid on-site evaluation. Subgroup data calculated specifically for patients requiring secondary procedure (*n* = 9).

**Table 7:** Univariate analysis of clinical, anatomical, and technical factors associated with tissue core adequacy.

Parameter	Inadequate Cohort (*N* = 7)	Adequate Cohort (*N* = 105)	*p*-Value
Age (years, Mean ± SD)	70.9 ± 6.5	66.8 ± 12.7	0.457
Body Weight (kg, Mean ± SD)	75.9 ± 14.5	76.1 ± 15.5	0.928
Pancreatic Lesion Size	<10 mm: 1 (14%)	<10 mm: 30 (29%)	0.671
	≥10 mm: 6 (86%)	≥10 mm: 75 (71%)	
ASA score	Class I: 2 (29%)	Class I: 20 (19%)	0.687
	Class II: 2 (29%)	Class II: 45 (43%)	
	Class III: 3 (43%)	Class III: 40 (38%)	
Lesion location	Head: 3 (43%)	Head: 34 (32%)	0.333
	Uncinate: 1 (14%)	Uncinate: 3 (3%)	
	Body: 2 (29%)	Body: 39 (37%)	
	Tail: 1 (14%)	Tail: 29 (28%)	
Needle tip	Franseen: 6 (86%)	Franseen: 84 (80%)	1.000
	Non-Franseen: 1 (14%)	Non-Franseen: 21 (20%)	
Needle Size	22G: 7 (100%)	22G: 103 (98%)	1.000
	20G: 0 (0%)	20G: 2 (2%)	
Endosonographer Experience	≤5 yrs: 6 (86%)	≤5 years: 52 (49.5%)	0.115
	>5 yrs: 1 (14%)	>5 years: 53 (50.5%)	
No. asses	<2: 3 (43%)	<2: 11 (10%)	0.041
	≥2: 4 (57%)	≥2: 94 (90%)	

SD, standard deviation; ASA, American Society of Anesthesiologists.

## Data Availability

The data presented in this study are available on request from the corresponding author due to institutional and privacy restrictions concerning patient-derived clinical and histopathological data.
